# Stoichiometry of Root and Leaf Nitrogen and Phosphorus in a Dry Alpine Steppe on the Northern Tibetan Plateau

**DOI:** 10.1371/journal.pone.0109052

**Published:** 2014-10-09

**Authors:** Jiangtao Hong, Xiaodan Wang, Jianbo Wu

**Affiliations:** 1 Institute of Mountain Hazards and Environment, Chinese Academy of Sciences, Chengdu, China; 2 University of Chinese Academy of Sciences, Beijing, China; Institute of Tibetan Plateau Research, China

## Abstract

Leaf nitrogen (N) and phosphorus (P) have been used widely in the ecological stoichiometry to understand nutrient limitation in plant. However,few studies have focused on the relationship between root nutrients and environmental factors. The main objective of this study was to clarify the pattern of root and leaf N and P concentrations and the relationships between plant nitrogen (N) and phosphorus (P) concentrations with climatic factors under low temperature conditions in the northern Tibetan Plateau of China. We conducted a systematic census of N and P concentrations, and the N∶P ratio in leaf and root for 139 plant samples, from 14 species and 7 families in a dry *Stipa purpurea* alpine steppe on the northern Tibetan Plateau of China. The results showed that the mean root N and P concentrations and the N∶P ratios across all species were 13.05 mg g^−1^, 0.60 mg g^−1^ and 23.40, respectively. The mean leaf N and P concentrations and the N∶P ratio were 23.20 mg g^−1^, 1.38 mg g^−1^, and 17.87, respectively. Compared to global plant nutrients concentrations, plants distributing in high altitude area have higher N concentrations and N∶P, but lower P concentrations, which could be used to explain normally-observed low growth rate of plant in the cold region. Plant N concentrations were unrelated to the mean annual temperature (MAT). The root and leaf P concentrations were negatively correlated with the MAT, but the N∶P ratios were positively correlated with the MAT. It is highly possible this region is not N limited, it is P limited, thus the temperature-biogeochemical hypothesis (TBH) can not be used to explain the relationship between plant N concentrations and MAT in alpine steppe. The results were valuable to understand the bio-geographic patterns of root and leaf nutrients traits and modeling ecosystem nutrient cycling in cold and dry environments.

## Introduction

Nitrogen (N) and phosphorus (P) are two essential elements in plants, both playing a critical role in plant function and most ecosystem processes [1]. A number of studies have reported species-specific differences (growth form, physiology, life history, etc.) and site-specific differences (temperature, precipitation, solar radiation, etc.) that account for much of the variability in plant-nutrient concentrations on large scales [2]–[6].

Temperature could be an important factor that drive the recirculation of nutrients in terrestrial ecosystems. Lower temperatures have depressing effect on microbial decomposition and mineralization of organic matter, which drastically reduce nutrient availability for plant [7], [8]. Furthermore, low-temperature supressing of nutrient uptake by root and soil nutrients migration are also well known phenomena [8]. Thus, Reich and Oleksyn [3] assumed that the global leaf N concentrations generally increased with the mean annual temperature (MAT) when the temperature was below 5–10°C (the temperature-biogeochemical hypothesis). Shi et al. [9] found the similar variation trend that leaf N concentrations increased with MAT under low temperature (MAT < 8.5°C) along an altitudinal gradient of Mount Gongga on the eastern Tibetan Plateau. However, other study reported that temperature had no effects on leaf concentrations, but phylogenetic variation was the key factor that affected the leaf N concentrations at the biome scale [5]. Wheather the temperature-biogeochemical hypothesis (TBH) could be used to explain the relationship between leaf N concentrations and MAT in alpine area is still a controversial issue on account of few plant data contained in previous studies [3], [4], [9]. In addition, the underlying mechanism of temperature on regulating leaf N in cold environment also deserves a further discussion.

Although ecological stoichiometry has been studied in terrestrial plants for many years, and most of studies focused on photosynthetic organs [10]–[12], we know little about the characteristics of root-nutrient stoichiometry along the climate gradient at a large scale, especially in cold and dry areas [13]–[15]. For example, Yuan et al. [15] compiled fine-root nitrogen and phosphorus data from 211 studies in 51 countries and reported that fine-root N∶P declined exponentially, but leaf N∶P decreased linearly with the increasing latitude (decreasing temperature) at global scale. However, only 11 species were compiled from tundra ecosystem in their study. The paucity of data limited not only the accurate evaluation of elements involved in biogeochemical cycles but also our understanding of plant nutrient concentrations response to the environmental factors under low temperature. Therefore, we need to provide a valuable contribution to the global data pool on root stoichiometry, given the previously limited knowledge on the tundra plant.

We studied the spatial patterns of leaf and root nutrients from 139 plant samples and their relationship with climatic variables at 32 sites distributed from east to west across the northern Tibetan Plateau. Our objectives were (1) to clarify the pattern of root and leaf N and P concentrations; (2) to quantify the relationship between plant nutrition and climatic factors, including MAT and mean annual precipitation (MAP) in a cold and dry climate.

## Materials and Methods

### Study sites

**Figure 1:**
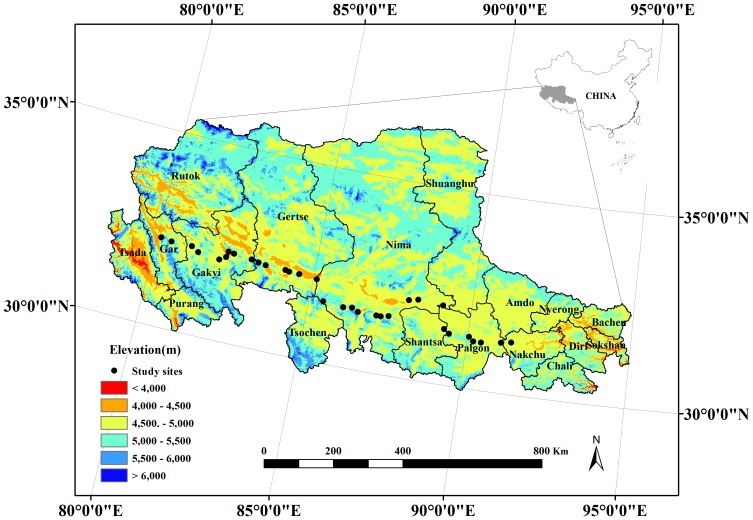
A map of 32 sampling sites of alpine steppe on the northern Tibetan Plateau.

The Tibetan Plateau, the highest plateau in the world, is extremely sensitive to climate change, and the vegetation has remained largely undisturbed by human activities; therefore, it is an important area to study global change ecology [16]. The alpine steppe in the northern Tibetan Plateau is a dry *Stipa purpurea* grassland with low precipitation, frequent gale-force winds, strong solar radiation and extensive permafrost. These have a profound effect on the soil nutrient status, the evolution of physiological processes and the adaptive mechanisms of plants [17], [18]. The study area was *Stipa purpurea* alpine steppe, located between 31.23°N–32.31°N and 80.12°E–91.35°E. The study sites were in seven counties (Nakchu, Palgon, Shantsa, Nima, Gerts, Gakyi and Gar) from east to west in the Tibet Autonomous Region of China with the similar soil type [19]. The sites were selected on flat terrain far from human habitats to minimise the influence of microtopography and grazing disturbance. Most sites were above 4500 m, with MAT below 0°C and MAP below 300 mm (Fig. 1; Table S1). The geographic information, such as latitude, longitude and altitude, of each site was recorded by a Global Positioning System (GPS) (Garmin, GPSMAP 62S, China).

### Plant sampling

We selected 32 sites along a belt transect (approx. 1300 km long) and collected 139 plant samples from 14 species and 7 families in August 2012 (Appendix). Each sample (15 cm length, 15 cm width, 30 cm depth) included the dominant species that was abundant at each site, and these samples were dug up using a spade. Then impurities on leaf and root surface were carefully cleaned. Individuals from the same species were combined, sun-dried in a paper envelope and brought back to the laboratory. In each sampling site, no specific permissions were required for collecting samples and the field studies did not involve endangered or protected species.

### Sample analysis

Plants were divided into roots and leaves, oven-dried at 65°C to a constant weight and ground into fine powder with a plant-sample grinder (TAISITE, High-speed Universal Disintegrator FW80, Tian Jin, China). The total N concentration was determined using the micro-Kjeldahl method after digesting the sub-samples in H_2_SO_4_-K_2_SO_4_-CuSO_4_ [20]. For the total P concentration determination, the sub-samples were digested in a H_2_SO_4_-HClO_4_ solution, and the P concentration was determined using phosphor molybdate blue spectrophotometry [20]. All the data were expressed as a mass (mg g^−1^).

### Climate data

The climate data from 1950 to 2000 for the sampling sites, including MAT and MAP, were obtained from the World Climate web site (www.worldclimate.com) with a resolution of 30″ (ca. 1 km) [4].

### Statistical analyses

The data of root and leaf N,P and N∶P ratio exhibited non-normal distribution in the present study. Therefore, the N and P concentrations and the N∶P ratios were log-transformed to meet normality for Pearson correlation and regression analysis and *T*-Tests, as is commonly done in analyses of plant N∶P stoichiometry [6], [9]–[11].The differences in the leaf N and P concentrations and N∶P ratios between the northern Tibetan Plateau and the other region were processed using independent sample *T*-Tests. The correlations of root and leaf N and P concentrations and N∶P ratios were analysed with regression analysis. The effects of the MAT and MAP on root and leaf N and P concentrations and N∶P ratios were tested by Pearson correlation analysis and simple regression analyses. All of the statistical analyses were performed with SPSS version 16.0 (SPSS Inc., USA), and cartograms were plotted using OriginPro 8.0 (OriginLab, MA).

## Results

### Patterns of N and P concentrations and the N∶P ratio in roots and leaves across all species

**Figure 2:**
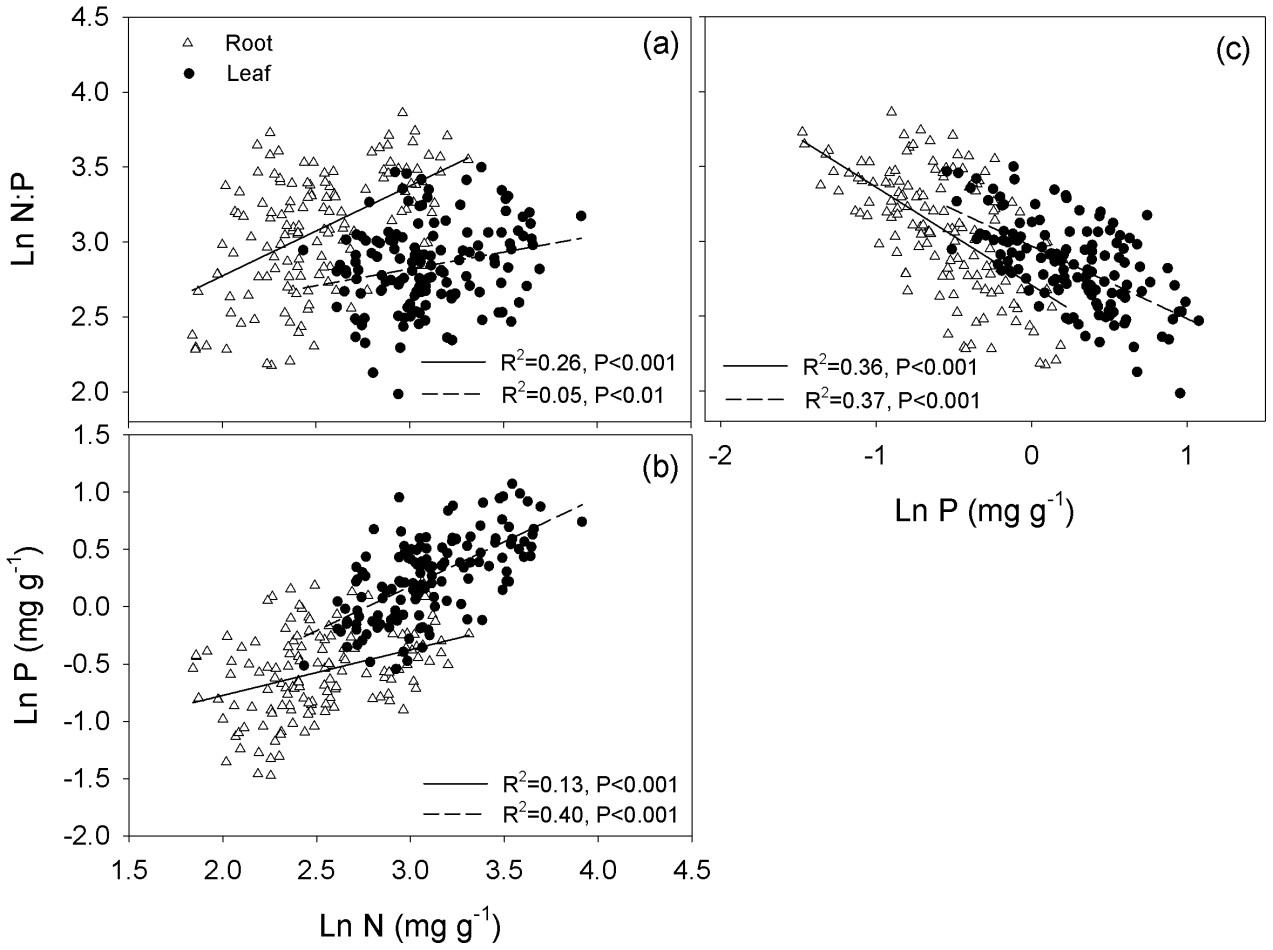
The relationship between the N and P concentrations (b) and the N∶P ratio (a, c) for roots and leaves across all species on the northern Tibetan Plateau. The solid line represents the root fitted straight line, the dashed line represents the leaf fitted straight line.

The mean values of root N and P concentrations and the N∶P ratio were 13.05 mg g^−1^, 0.60 mg g^−1^ and 23.40 (Table 1). The average values of leaf N and P concentrations and the N∶P ratio were 23.20 mg g^−1^, 1.38 mg g^−1^ and 17.87, respectively (Table 1). The root and leaf N and P concentrations were positively correlated with each other (root: *R^2^* = 0.13, *P*<0.001; leaf: *R^2^* = 0.40, *P*<0.001, see Fig. 2b). The root and leaf N∶P ratios were more strongly correlated with P concentrations (root: *R^2^* = 0.36, *P*<0.001; leaf: *R^2^* = 0.37, *P*<0.001) than with N concentrations (root: *R^2^* = 0.26, *P*<0.001; leaf: *R^2^* = 0.05, *P*<0.01, see Fig. 2a, c).

**Table 1:** Root and leaf N, P concentrations and N∶P ratios for the overall plant in northern Tibet Plateau.

	Organ	*n*	N (mg g^−1^)			P (mg g^−1^)			N∶P		
			Mean	SD	CV	Mean	SD	CV	Mean	SD	CV
Overall	Root	139	13.05	4.67	0.36	0.60	0.22	0.37	23.40	8.62	0.37
	Leaf	139	23.20	7.19	0.31	1.38	0.50	0.36	17.87	5.21	0.29

**Figure 3:**
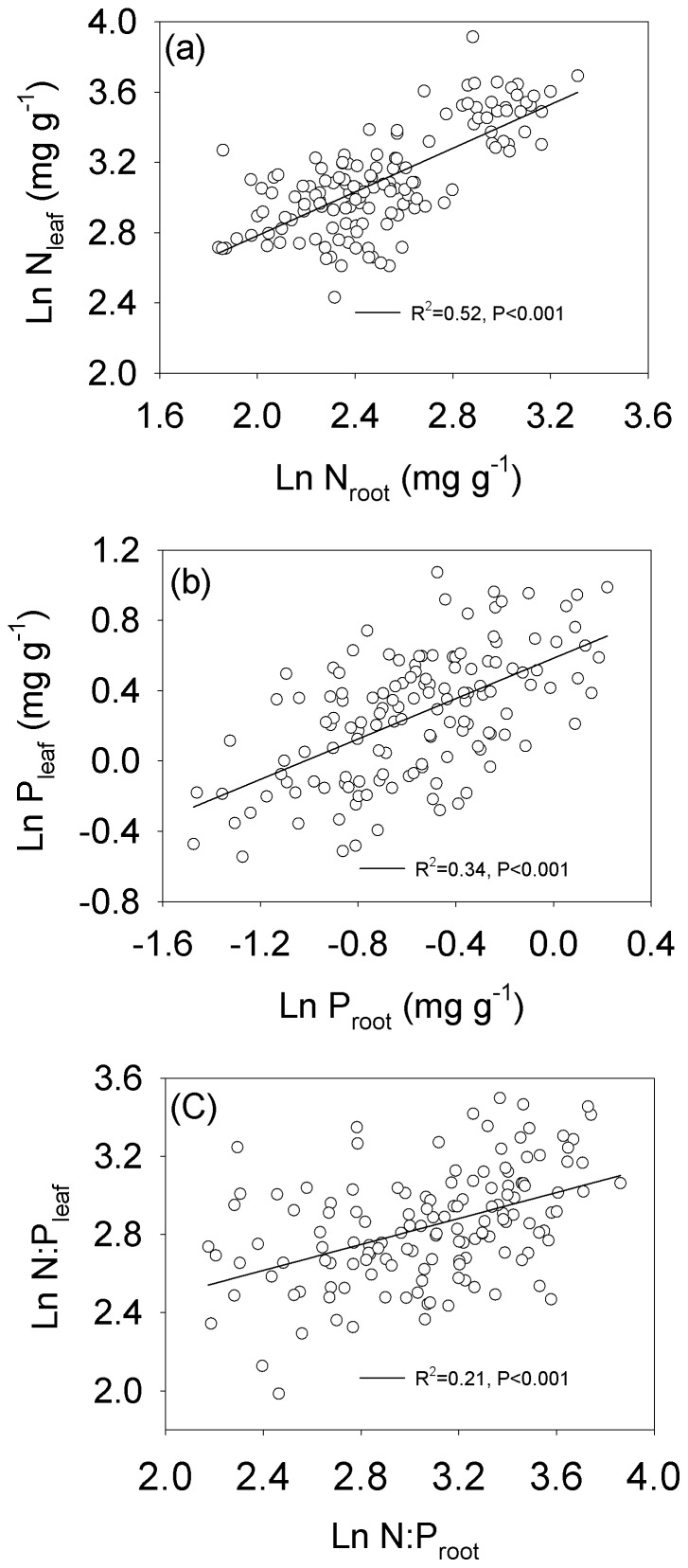
The relationship between the root and leaf N∶P stoichiometry across all species on the northern Tibetan Plateau.

Root N and P concentrations and N∶P ratio were positively correlated with leaf N and P concentrations and N∶P ratio, respectively. (N: *R^2^* = 0.52, *P*<0.001; P: *R^2^* = 0.34, *P*<0.001; N∶P: *R^2^* = 0.21, *P*<0.001, see Fig. 3a, b, c).

### Relationship of N and P concentrations and the N∶P ratio in roots and leaves with climate factors

**Figure 4:**
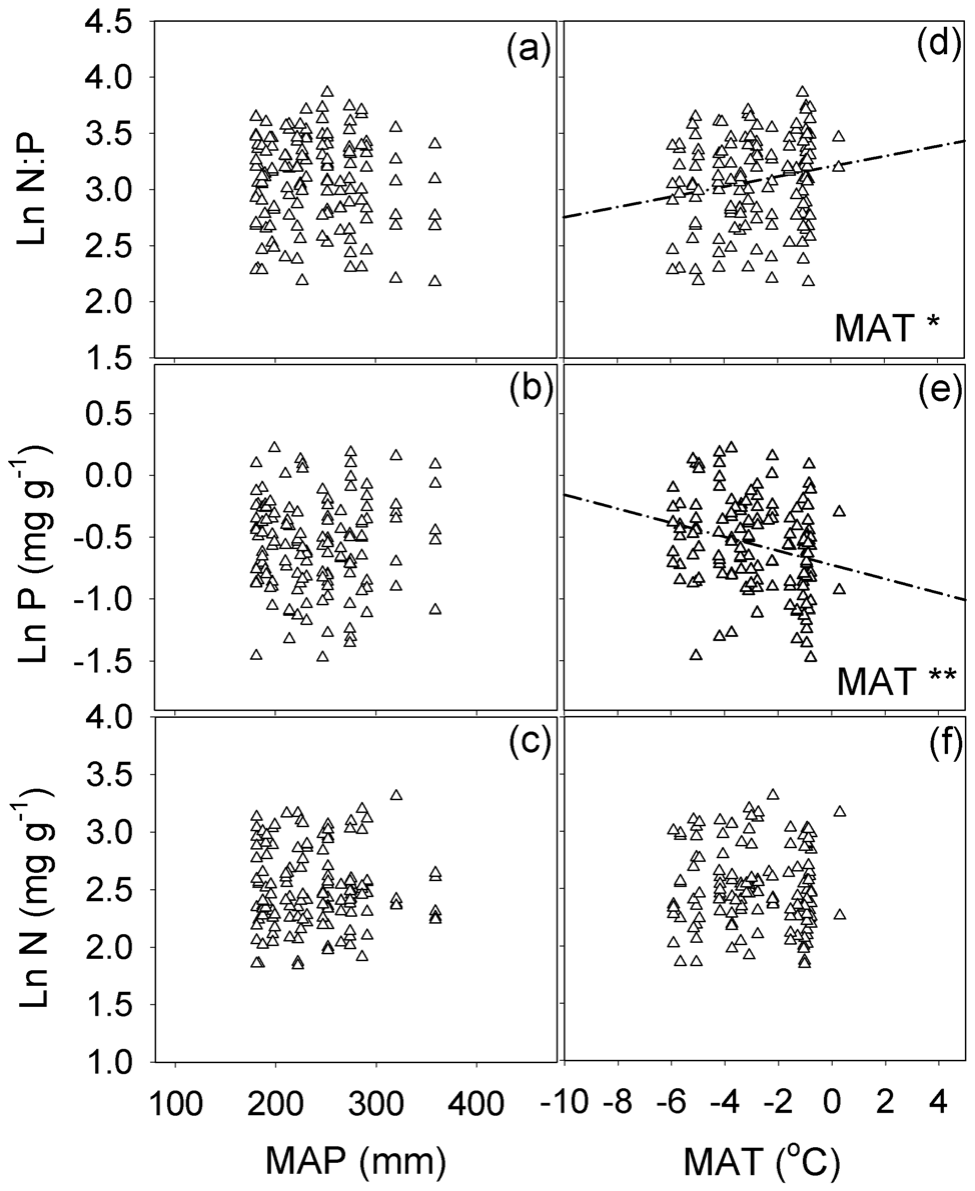
The relationship between the root N∶P stoichiometry and the MAP and MAT across all species on the northern Tibetan Plateau. Regression lines are shown only for relationships that were significant at *P*<0.05. * and ** represent relationships that significant at the 0.05 and 0.01, respectively.

**Figure 5:**
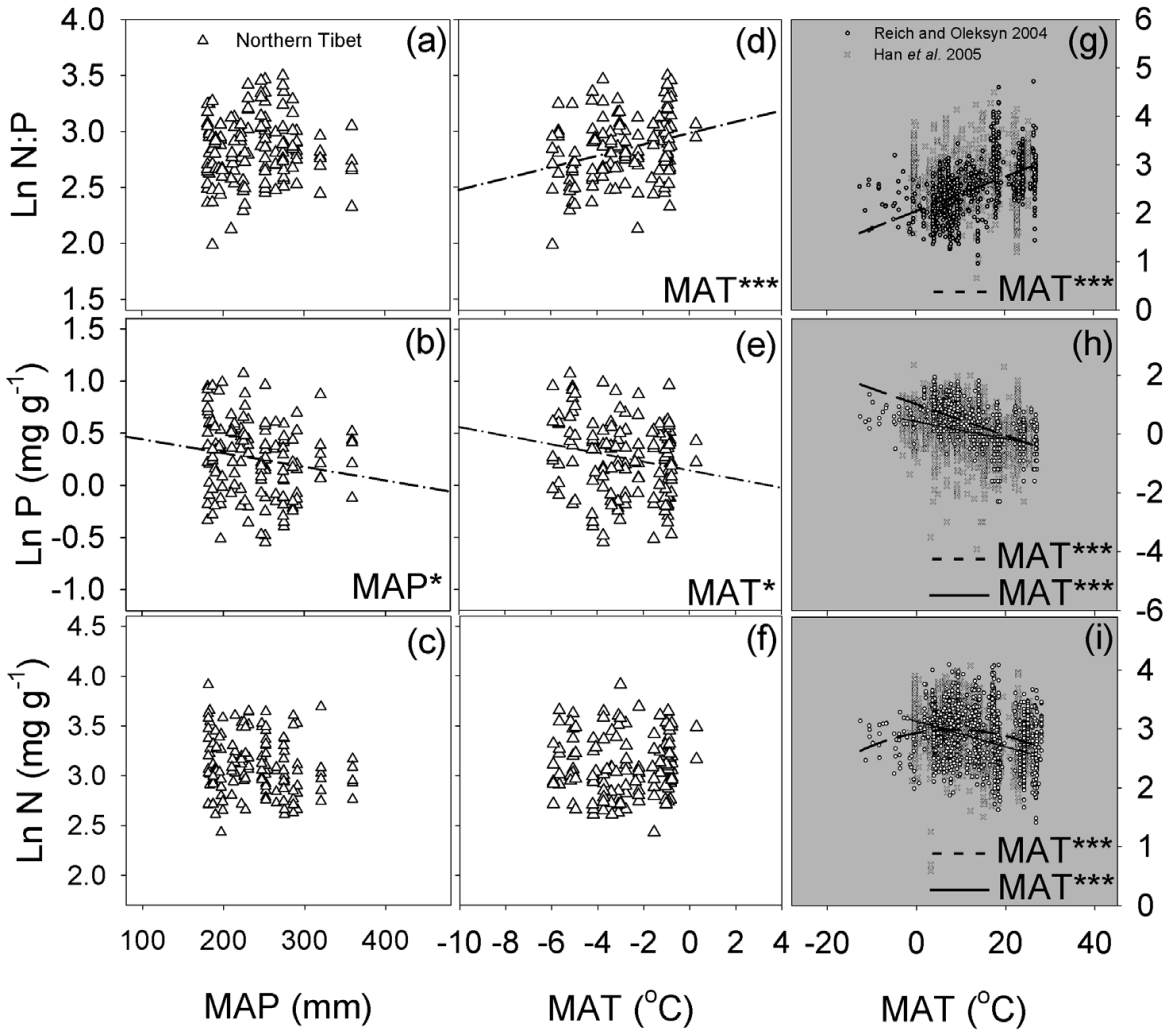
The relationship between the leaf N∶P stoichiometry and the MAP and MAT of the northern Tibetan Plateau (a–f), China and global (g–i). The dashed dot line represents the fitted straight line of the leaf N∶P stoichiometry with the MAT and MAP on the northern Tibetan Plateau, the dashed line represents the fitted straight (curved) line of the global leaf N∶P stoichiometry with MAT from Reich and Oleksyn (2004), the solid line represents the fitted straight line for Chinese leaf stoichiometry N∶P with the MAT from Han et al. (2005). Regression lines are shown only for relationships that were significant at *P*<0.05. * and *** represent relationships that significant at the 0.05 and 0.001, respectively.

Across all species, both the root and leaf N concentrations were not significantly correlated with the MAT and MAP (*P*>0.05), while the root and leaf P concentrations were negatively correlated with the MAT [root: correlation coefficient (*r*) = −0.26, *P*<0.01; leaf: *r* = −0.20, *P*<0.05] (Table 2). Positive linear correlations were detected between the N∶P ratio and the MAT for roots and leaves (root: *r* = 0.19, *P*<0.05; leaf: *r* = 0.30, *P*<0.001). However, the root and leaf N∶P ratio had no significant relationship with the MAP (*P*>0.05). In addition, the leaf P concentration was negatively correlated with the MAP (*r* = −0.17, *P*<0.05) but not with the root P concentration (*P*>0.05) (Table 2, Fig. 4, Fig. 5).

**Table 2:** Correlation coefficients between plant N, P, and N∶P ratio, and climate factors (MAT, MAP) in northern Tibet Plateau.

Organ		MAT	MAP	Organ		MAT	MAP
Root	N	−0.06	−0.05	Leaf	N	0.05	−0.16
	P	−0.26**	0.03		P	−0.20*	−0.17*
	N∶P	0.19*	−0.07		N∶P	0.30***	0.05

*, **, and *** represent correlation that is significant at the 0.05, 0.01 and 0.001 level (2-tailed), respectively.

## Discussion

### Patterns of root and leaf N and P concentrations and the N∶P ratio across all species

In this study, the mean root N concentration of the 139 samples was higher than that reported for the Inner Mongolia grassland of China by Zhou et al. [14] and the global data reported by Yuan et al. [15] (Table 3). However, the root P concentration of plants in northern Tibet was slightly lower than that of the Inner Mongolia grassland of China [14] but much lower than global data [15]; as a result, the root N∶P ratio in the northern Tibetan Plateau was 50% higher than that of Inner Mongolia species [14] and 46% higher than the average global data [15] (Table 3). The leaf N concentration in our samples was significantly higher than that found in 753 Chinese plant species by Han et al. [21] and in 1251 global terrestrial plant species by Reich and Oleksyn [4], but lower than that of the Chinese grassland species [6]. Our sampled plant species had lower leaf P concentrations than those of the Chinese grassland [6] and the global averages [4], but our values were slightly lower than that of the Chinese flora [21] The leaf N∶P ratios were 9%, 17% and 29% higher than those in the Chinese flora [21], the Chinese grassland [6] and the global vegetation [4] (Table 3), respectively. In conclusion, the northern Tibetan Plateau samples had a higher N concentration and N∶P ratio, but a lower P concentration.

**Table 3:** The mean and range of root and leaf N, P and N∶P ratio in this study and others.

Data source	N (mg g^−1^)		P (mg g^−1^)		N∶P		References
	Mean	Range	Mean	Range	Mean	Range	
Root							
Northern Tibet	13.05	6.30–27.42	0.60	0.23–1.25	23.40	8.80–47.55	This study
Inner Mongolia Grassland	10.90	—	0.7	—	15.57	—	(Zhou et al. 2010)
Global study	9.8	—	0.78	—	16.0	—	(Yan et al. 2011)
Leaf							
Northern Tibet	23.20	11.39–50.13	1.38	0.58–2.93	17.87	7.28–33.09	This study
Chinese grassland	27.6	—	1.9	—	15.3	—	(He et al. 2008)
Chinese flora	20.24***	6.25–52.61	1.45**^NS^**	0.05–10.27	16.35**^NS^**	3.28–78.89	(Han et al. 2005)
Global flora	20.1***	4.1–59.9	1.77***	0.1–6.99	13.8***	2.6–111.8	(Reich and Oleksyn 2004)

*** Significant differences in leaf N and P concentrations and the N∶P ratio between the northern Tibetan Plateau and other areas at *P*<0.001, NS represents no significant difference in the leaf P content and the N∶P ratio between the northern Tibetan Plateau and other areas (*P*>0.05).

It is well known that N and P are the major nutrients constraining plant growth throughout the world [22], [23]. Koerselman and Meuleman [24] showed that a leaf N∶P ratio of 14 indicates an N limitation, while a ratio of 16 indicates a P limitation. The mean value of the leaf N∶P ratio in this study was 17.87, which indicated that the plant in the alpine steppe of the northern Tibetan Plateau was more limited by P, as was shown in the Chinese flora analysed by Han et al. [21]. One probable reason for this result is the lower soil P content across China compared with the global average [25], and generally plant P concentration was affected by soil P within the ecosystem [21]. Moreover, Tian et al. [26] found that the soil P content in this frigid highland was even lower than that of the Chinese average. Because most P input is deposited on the soil surface by weathering, but gale-force winds (wind speed > 17.2 m • s^−1^) occurred on more than 100 days per year in this area [27]; thus, the nutrient was easily migrated and lost due to low plant cover and the intense erosion effects of wind [28]–[31]. In addition, the soil on the alpine steppe is alkaline, with a high soil pH and an abundance of CaCO_3_ that leads to low P bioavailability [32].

The leaf N concentration in the cold northern Tibetan Plateau was significantly higher than the result reported by Han et al. [21] and Reich and Oleksyn [4] (Table 3). The higher N concentration of high-altitude plants may be a result of the “inherent developmental growth constraints inhibiting nutrient dilution in the plant body” [33]. According to Reich and Oleksyn [4], the high leaf N concentration offsets the inefficiency of enzymes and the physiological processes in cold environment, thereby increasing the metabolic efficiency of plants. At most of our sampling sites with an MAP below 300 mm and many extremely windy days, intense evaporation increased the water stress during soil nitrogen mineralisation and plant growth. This may explain why the leaf N concentration in alpine steppe was lower than other grassland types of China [5], a result of allocating more N to the root to increase the water absorption capacity with increasing drought [34], [35]. Thus, the root N concentration on the northern Tibetan Plateau was higher than that in the Inner Mongolia grassland of China [14] and higher than global averages [15].

Across all species, the CVs of root N and P concentrations and the N∶P ratio at our sites were larger than those in the leaves, indicating that the leaf stoichiometry was more stable than that of the root (Table 1). The complexity of the root may play a key role because they have more multistage branches and the structure and function of each branch is quite different [36]. Root N and P concentrations were generally less than half of the leaf concentrations in this study (Table 1), because leaf was the most important photosynthetic organ requiring high nutrient concentrations to improve the plant's photosynthetic and metabolic capacity [37]. The N and P concentrations were strongly correlated for the roots and leaves (Fig. 2. b), and the relationships observed in this study were similar to those reported by Wright et al. [38].

Higher leaf nutrient concentrations were accompanied by high root nutrient concentrations in present study (Fig. 3. a, b). Roots belong to the “structural” component and leaves belong to the “metabolic” component. The nutrient-rich leaves always exhibited high photosynthetic and metabolic activity, required corresponding higher nutrient investments in “structural” tissue [37]. Because higher nutrient concentrations in “structural” tissue may lead to high rates of nutrient recycling in the phloem, which is linked closely with increased photosynthate export and phloem loading [39].

### Relationship between the N and P concentrations and the N∶P ratio in roots and leaves with climate factors

**Figure 6:**
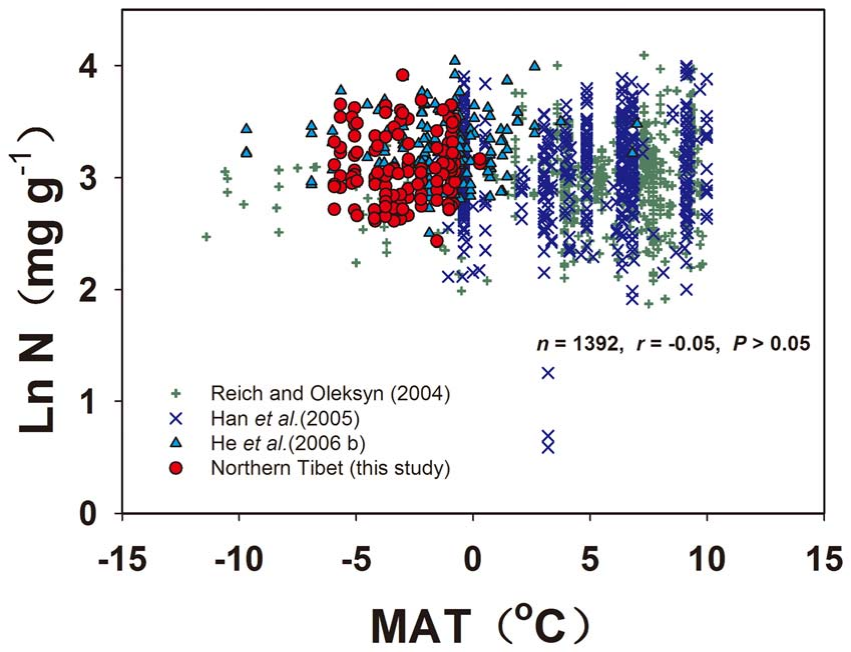
The relationship between leaf N and the MAT under low temperatures (MAT <10°C), including northern Tibetan Plateau data (this study), global data from Reich and Oleksyn (2004), Chinese data from Han et al. (2005) and eastern Tibetan Plateau data from He et al. (2006 b).

According to Reich and Oleksyn [4], the leaf N concentration increased with the MAT when the temperature was below 5–10°C, because a low MAT limited soil N mineralization, and reduced soil availability and root-nutrient absorption. However, in this study, the leaf N concentration of all species in the northern Tibetan Plateau was unrelated to the MAT (Fig. 5. f). We assumed that the temperature had no effect on leaf N in cold regions with an MAT below 0°C, which was consistent with the viewpoint of He et al. [5]. Even when we combined our leaf N data, the global data from Reich and Oleksyn [4], the Chinese data from Han et al. [21] and the eastern Tibetan Plateau data from He et al. [16] with MAT below 10°C together, no relationship was observed between the leaf N concentration and the MAT in cold regions (*n* = 1392, *r* = −0.05, *P*>0.05) (Fig. 6). Why was the leaf N concentration not sensitive to an increasing MAT under low temperatures? First, He et al. [5] argued that phylogenetic variation (genus) was the key factor that affected the leaf N concentrations at the biome scale, not climate conditions. Second, a warming climate may have no effect or a relatively small effect on soil N availability in alpine and arctic environments during the growing season [40]–[44]. A warming climate may result in abundant nutrients draining from the thick layer of permafrost by means of soil water movement (especially in the fallow season) due to intense freeze-thaw cycles in alpine tundra [45]–[47]. Third, in cold terrestrial ecosystems, low temperature is generally considered a limiting factor controlling plant growth. Even if the rising rate of soil N mineralisation could improve root absorption ability with the temperature increasing, but the increased biomass could dilute the extra absorption of nitrogen to maintain leaf N concentrations relatively stable [48], [49].

The leaf P concentration was negatively correlated with the MAT, which was the same trend observed by Reich and Oleksyn [4] and Han et al. [21] (Fig. 5. e, h). A negative relationship between the leaf P concentration and the MAP was found in this study (Fig. 5. b). The higher precipitation and temperature could result in higher rate of rock weathering and more P leaching from the soil [50], [51]. Additionally, due to the inhibiting influence of cold-adapted phosphatases activities with increasing temperatures, P immobilisation would increase more rapidly with temperature than P mineralization; therefore, the dissolved inorganic P released from alpine and tundra soils was significantly greater at lower temperature [52], [53]. The variation of the leaf P concentration across the temperature gradient resulted in leaf N∶P ratio was positively correlated with the MAT, which was consistent with the result of Reich and Oleksyn [4] (Fig. 5. d, g). Nevertheless, all the simple linear regression line in our study have gender slopes than those reported by Han et al. [21] and Reich and Oleksyn [4]. This may be caused by the smaller extent of MAT on the northern Tibet Plateau.

Yuan et al. [15] reported a different pattern of leaf and root N∶P with the latitude (temperature changes) at global scale. Root N∶P declined exponentially, but leaf N∶P decreased linearly with the increasing latitude (decreasing temperature). However, in present study, the root P concentrations had a negatively linear correlation with the MAT, and the root N: P ratio also had a positively correlation with the MAT, which was similar to the result for with leaves (Fig. 4. d, e; Fig. 5. d, e). Further studies will be required before the conclusions could be made about the differences or similarities in plant-climate biogeographical variation in root and leaf stoichiometry.

## Conclusions

We conclude from our observations that correlations exist between root and leaf N and P with climate factors (MAP and MAT), even in a particular arctic/alpine environment such as the northern Tibetan Plateau, which indicates similar evolutionary strategies and phylogenetic signals to plants in other regions. However, the temperature-biogeochemical hypothesis (TBH) can not be used to explain the relationship between plant N concentrations and MAT in alpine steppe. Our study also provided a valuable contribution to the global data pool on root stoichiometry, given the previously limited knowledge on the alpine plant. Understanding the bio-geographic patterns of root and leaf nutrients concentrations and the relationships with climatic factors will aid in modeling ecosystem nutrient cycling and predicting plant functional traits response to global changes in high altitude area.

## Supporting Information

Table S1 **Positional information and climate data for the 32 sites where plant samples were collected.**
(DOC)
