# Supplementary material for: Stoichiometry of Root and Leaf Nitrogen and Phosphorus in a Dry Alpine Steppe on the Northern Tibetan Plateau
Source: PLoS One. 2014 Oct 9;9(10):e109052. doi: 10.1371/journal.pone.0109052 (PMC4192305; doi:10.1371/journal.pone.0109052)
Supplement: Table S1 — Positional information and climate data for the 32 sites where plant samples were collected. (DOC) [file pone.0109052.s001.doc]

**Supporting Information**

**Table S1 Positional information and climate data for the 32 sites where plant samples were collected.**

| County | Site location | | | Weather conditions | | Sampling species |
| --- | --- | --- | --- | --- | --- | --- |
|  | Latitude  (^o^N) | Longitude  (^o^E) | Altitude  (m) | MAT  (^o^C) | MAP  (mm) |  |
| Nakchu | 31.34 | 91.35 | 4565 | -0.84 | 359 | Sp,Cm,Ln,Pb,Ss |
| Palgon | 31.30 | 91.04 | 4929 | -2.21 | 320 | Sp,Ln,Og,Pb,Ys,Ic |
| Palgon | 31.23 | 90.43 | 5002 | -2.78 | 291 | Sp,Cm,Ln,Og,Pb,Ss,Ys |
| Palgon | 31.23 | 90.19 | 5297 | -4.19 | 275 | Sp,Cm,Ln,Og,Aw,Ic,Lb |
| Palgon | 31.33 | 90.04 | 4887 | -3.10 | 286 | Sp,Cm,Ln,Og,Om,Pb |
| Palgon | 31.33 | 89.42 | 5118 | -3.41 | 265 | Sp,Cm,Ln,Pb,Ss |
| Palgon | 31.43 | 89.25 | 5163 | -3.74 | 252 | Sp,Cm,Ln,Og,Pb,Aw |
| Shantsa | 32.02 | 89.11 | 4560 | -0.93 | 274 | Sp,Cm,Ln,Og,Pb,Ys,Lb,Om |
| Nima | 32.06 | 88.32 | 4631 | -1.06 | 252 | Sp,Cm,Ln,Og,Ss,Ic |
| Nima | 32.01 | 88.04 | 4609 | -0.78 | 247 | Sp,Cm,Ln,Og,Pb,Os,Ic |
| Nima | 31.52 | 87.50 | 4776 | -0.94 | 231 | Sp,Cm,Og,Pb,Os,Om |
| Nima | 31.48 | 87.27 | 4758 | -1.03 | 222 | Sp,Cm,Ln,Og,Pb |
| Nima | 31.48 | 87.13 | 4795 | -1.29 | 214 | Sp,Cm,Ln,Og,Pb, |
| Nima | 31.48 | 86.55 | 5561 | -5.67 | 183 | Sp,Cm,Ln,Og,Pb,Lb,Om |
| Nima | 31.56 | 86.35 | 5467 | -5.07 | 181 | Sp,Cm,Og,Pb,Os,Om |
| Nima | 31.53 | 86.08 | 4851 | -1.55 | 197 | Sp,Cm,Ln,Og,Pb,Om |
| Nima | 31.58 | 85.44 | 5289 | -5.93 | 187 | Sp,Cm,Ln,Og,Pb |
| Gerts | 31.88 | 84.89 | 5230 | -3.38 | 190 | Sp,Om,Aw |
| Gerts | 32.06 | 84.51 | 5115 | -3.60 | 191 | Sp |
| Gerts | 32.15 | 84.23 | 5265 | -4.08 | 191 | Sp,Om |
| Gerts | 32.17 | 84.10 | 5022 | -3.20 | 195 | Sp,Aw |
| Gerts | 32.18 | 83.47 | 4398 | 0.29 | 222 | Sp,Om |
| Gerts | 32.21 | 83.24 | 4856 | -2.36 | 212 | Sp |
| Gerts | 32.25 | 83.01 | 4887 | -2.76 | 211 | Sp,Om |
| Gakyi | 32.29 | 82.44 | 4808 | -2.23 | 210 | Sp,Aw |
| Gakyi | 32.31 | 82.26 | 4748 | -1.62 | 210 | Sp |
| Gakyi | 32.16 | 82.22 | 5389 | -5.18 | 225 | Sp,Pb,Ot,Om |
| Gakyi | 32.06 | 82.03 | 4600 | -0.88 | 252 | Sp,Os,Om,Ot |
| Gakyi | 32.12 | 81.34 | 5272 | -4.96 | 227 | Sp,Pb,Os,Ot |
| Gakyi | 32.23 | 81.11 | 5039 | -3.75 | 199 | Sp,Os,Ot |
| Gar | 32.22 | 80.46 | 4887 | -3.01 | 181 | Sp,Og,Om |
| Gar | 32.26 | 80.12 | 5084 | -4.22 | 187 | Sp,Ay |

Sampling species included *Stipa purpurea* (Sp)*, Carex moorcroftii* (Cm)*, Leontopodium nanum* (Ln)*, Oxytropis glacialis* (Og)*, Potentilla bifurca* (Pb)*, Oxytropis serioopetala* (Os)*, Youngia simulatrix* (Ys)*, Incarvillea compacta* (Ic)*, Lagotis brachystachya* (Lb)*,Oxytropis microphylla* (Om)*, Artemisia wellbyi* (Aw)*, Artemisia younghusbandii* (Ay) *Orinus thoroldii* (Ot) and *Saussurea stoliczkai* (Ss)*.*

Financial disclosure information have been deleted in the manuscript.The funders had no role in study design, data collection and analysis, decision to publish, or preparation of the manuscript.No graphics or color was included in the manuscript or legends.We have removed figure insertion instructions from the manuscript.
